# Genetic testing for *BRCA1/2* variants in Northern African women with ovarian and breast cancers: a multicentre study of an under-represented ancestry

**DOI:** 10.1016/j.esmoop.2025.105510

**Published:** 2025-07-25

**Authors:** K.S. Shohdy, L. Kassem, T. Elnahas, E.M. Barsoum, B. Gabriel, H. Elghazawy, S. Lasheen, C. Gourley, H.A. Azim

**Affiliations:** 1 School of Cancer Sciences and Cancer Research UK Scotland Institute, University of Glasgow, UK; 2 Department of Clinical Oncology, Cairo University, Cairo, Egypt; 3 Barsoum Oncology Center, Giza, Egypt; 4 Nasser Institute for Research and Treatment, Ministry of Health and Population, Cairo, Egypt; 5 Department of Clinical Oncology, Ain Shams University, Cairo, Egypt; 6 Nicola Murray Centre for Ovarian Cancer Research, Cancer Research UK Scotland Institute, Institute of Genetics and Cancer, University of Edinburgh, Edinburgh, UK

**Keywords:** *BRCA1/2*, pathogenic variants, ovarian cancer, breast cancer, Northern African

## Abstract

**Background:**

There is a lack of studies investigating the burden of *BRCA1/2* pathogenic variants (PVs) in Northern African countries using next-generation sequencing (NGS)-based testing in patients with epithelial ovarian (EOC) and triple-negative breast cancer (TNBC).

**Patients and methods:**

We established a multicentre registry for genetic testing of unselected patients referred from five centres from 2019 to 2022 across Egypt. Germline or somatic *BRCA1/2* sequencing was carried out by target enrichment using the AmoyDx® *BRCA1*/*2* Mutation Kit, and sequencing was carried out using the Illumina NextSeq500 system.

**Results:**

Genetic testing was successfully carried out for 1349 of 1420 (95%) patients tested (EOC = 1031, TNBC = 318). The median age was 55 years (range 20-86 years). We identified 258 *BRCA1/2* PVs affecting 254 (18.8%) patients. The rate of *BRCA1/2* PVs in EOC and TNBC was 18.2% and 20.8%, respectively. Although the rate of PVs in our cohort was similar to the Western cancer population, there were significant differences in the enrichment at the variant level. The majority of recurrent *BRCA2* PVs, but not *BRCA1* PVs, were breast or ovarian lineage-unique. We developed a limited-resource conscious framework that provided a reclassification of 60% of identified variants of uncertain significance (VUS).

**Conclusion:**

Our findings would help to enhance cancer care and enrich the genetic epidemiology of women from such understudied ancestries.

## Highlights

• Central NGS testing was feasible in resource-limited settings, with rates similar to those observed in Western populations.
• Women of Northern African ancestry exhibited unique *BRCA1/2* variation patterns that differed from other ancestries.
• A resource-conscious bioinformatic framework enabled reclassification of 60% of the identified VUS.

## Introduction

Pathogenic variants (PVs) in *BRCA1/2* in patients with ovarian and triple-negative breast cancer (TNBC) were extensively studied in the Western population. However, there is a paucity of studies investigating the burden of *BRCA1/2* in Northern African countries using next-generation sequencing (NGS)-based genetic testing. Suboptimal treatment and poorer survival rates have been reported in low-income countries.^1^ Recent advancements in genomic research have identified the crucial role of *BRCA* gene mutations in predisposing individuals to hereditary breast and ovarian cancers.^2^^,^^3^ The lifetime risk of developing breast or ovarian cancer for *BRCA1* carriers is 72% and 44%, respectively and for *BRCA2* carriers is 69% and 17%, respectively.^4^ The presence of *BRCA* mutations can profoundly impact disease management and treatment outcomes, necessitating a comprehensive understanding of the clinical landscape surrounding these specific patient populations. For instance, specific *BRCA* mutations can mediate different disease phenotypes.^5^

Several studies have reported substantially diverse findings on the rate of *BRCA* mutations in Egyptian women,^6^, ^7^, ^8^ underscoring the need to refine evidence regarding the true prevalence of *BRCA* in the Egyptian population and the clinical implications. Our group recently conducted a systematic review of the literature that revealed a number of inconsistencies in the germline *BRCA* (g*BRCA*) testing for Egyptian women with breast cancer, including an unexpectedly high mutation rate that could indicate issues with the validity of the tests.^9^ Our study aimed to address these gaps in knowledge by conducting a large-scale registry study to investigate the genetic landscape of patients with breast and ovarian cancer. We sought to define the true prevalence of *BRCA* PVs in patients using whole-gene NGS analysis and a combined bioinformatic framework that reclassifies the variants of uncertain significance (VUS) in view of the lack of genetic epidemiology, case-control and phenotype data from such understudied ancestry. In addition, we conducted extensive comparisons with the data from multiple Western populations to identify unique *BRCA* variations in the Northern African ancestry.

## Methods

### Patient enrolment and tissue acquisition

All experimental procedures were carried out in accordance with approved guidelines and were approved by the Institutional Review Boards at Dar Salam Cancer Centre (Ministry of Health and Population-Egypt No. 2-2024/20). This was a multicentre prospectively maintained registry study that included patients referred from five cancer centres from September 2019 to August 2022 across Egypt. Eligible patients were diagnosed with histologically confirmed TNBC (for which *gBRCA1/2* testing was carried out) or newly diagnosed ovarian, fallopian tube or primary peritoneal epithelial cancer (hereafter referred to as epithelial ovarian cancer, EOC) where germline or somatic *BRCA1/2* testing was carried out.

### Molecular testing

For somatic *BRCA1/2* testing, sequencing was carried out on any relevant tissue sample containing an adequate (>30%) proportion of neoplastic cells. Processed formalin-fixed, paraffin-embedded (FFPE) tissue samples and histological sections mounted on unstained glass slides <12 months old were utilized for nucleic acid extraction. Germline testing was carried out using whole blood collected in EDTA tubes. DNA was extracted from FFPE tissue or peripheral blood sample using QIAamp DNA Mini kit (QIAGEN, Hilden, Germany). For FFPE and paired peripheral blood samples, a minimum DNA yield of 50 ng was required. *BRCA1/2* NGS was carried out by target enrichment using the AmoyDx® (AmoyDx®, Xiamen, China) *BRCA1* and *BRCA2* Gene Mutation Detection Kit, and sequencing was carried out using the Illumina NextSeq500 system (Illumina, San Diego, CA). Testing included full gene sequencing and was able to identify single nucleotide variants (SNV), insertions, deletions (InDel) and large gene rearrangements (LRs). The analysis covered all coding exons, the border regions between exons and introns, some introns, and untranslated regions (UTR) of the *BRCA1* and *BRCA2* genes.

Mutations were called if the following criteria were met: for blood samples, a raw sequencing depth of ≥100×, a mutant allele frequency ≥20%; for fresh tissue and FFPE samples, a raw sequencing depth of ≥300×. Bioinformatic analysis was carried out using the AmoyDx NGS data analysis system software (ANDAS) (AmoyDx®, Xiamen, China) to obtain the related gene variant information. Interpretation of pathogenicity followed the American College of Medical Genetics and Genomics (ACMG) guideline.^10^ All mutations and genetic variants were referenced to cDNA positions on their respective primary transcripts and named according to the Human Genome Variation Society (HGVS) convention.

The primary endpoint was the prevalence of *BRCA1/2* pathogenic variants in Egyptian women with EOC and TNBC. Secondary endpoints included the comparison of the Egyptian PVs and VUS landscape against the publicly available Western populations, in addition to the attempt to reclassify the VUS detected in our series using genomic databases, population data, experimental/functional data and computational prediction tools.

### Comparison with the MSK cohort

Clinical and genetic data of the Memorial Sloan Kettering (MSK) EOC cohort (*n* = 384) were extracted from the supplementary tables.^11^ All patients underwent prospective sequencing as part of their clinical care using the MSK-IMPACT targeted sequencing panel. Genomic sequencing was carried out on tumour DNA extracted from FFPE and germline DNA was sequenced in all patients. Pathogenicity assessment of *BRCA1/2* followed the ACMG, as described in the literature, Richards et al.^10^

### Comparison with the UK diagnostic laboratory cohort

Data on cases who underwent NGS testing for *BRCA1/2* at 1 of the 25 molecular diagnostic laboratory and clinical genetics services of the UK (National Health Service) and Ireland were accessible through the Can-Var UK portal.^12^^,^^13^

### Reclassification of VUS

We conducted a systematic review of the literature for experimental annotations of our VUS and adopted two robust computational tools, the MVP (Missense Variant Pathogenicity prediction) for missense variants^14^ and SpliceAI for intron variants.^15^ High-quality experimental data were defined by the cancer variant interpretation group (Can-VIG UK).^13^ MVP uses a deep residual network for accurate prediction of missense pathogenicity and outperformed all other methods with an area under the curve (AUC) of 0.96 in an independent cohort. Code for missense prediction is available at https://github.com/ShenLab/missense. Variants with MVP scores of ≥0.85 were considered pathogenic. Variants with a score <0.70 were considered benign, and variants with a score between 0.70 and 0.85 were considered VUS.

To predict the pathogenicity of intronic VUS, we ran the SpliceAI pipeline. This package annotates genetic variants with their predicted effect on splicing, as described by Jaganathan et al.^15^ We used the default parameters including a 500 bp window. The code is available through https://github.com/Illumina/SpliceAI. Variants with a maximum delta score <0.10 are considered benign and variants with a score >0.20 are considered candidate pathogenic. Variants within 0.10-0.20 remained unclassified as VUS.

### Statistical analysis

Comparisons of frequencies were analysed based on variable categories with the chi-square or Fisher’s exact test. A comparison of numeric variables was carried out using the Mann–Whitney *U* test. Logistic regression models and odds ratio (OR) were used to estimate the association between predictor variables and binary outcomes. A *P* value <0.05 was considered significant. Statistical analyses were conducted with STATA 14.1 (StataCorp, College Station, TX) software and Rstudio (v2024.12.1, Posit Software, PBC, Boston, MA). For gene and variant visualizations, the StJude ProteinPaint and cbioportal tools were used to create oncoplots.

## Results

### Overview of the study

**Figure 1:**
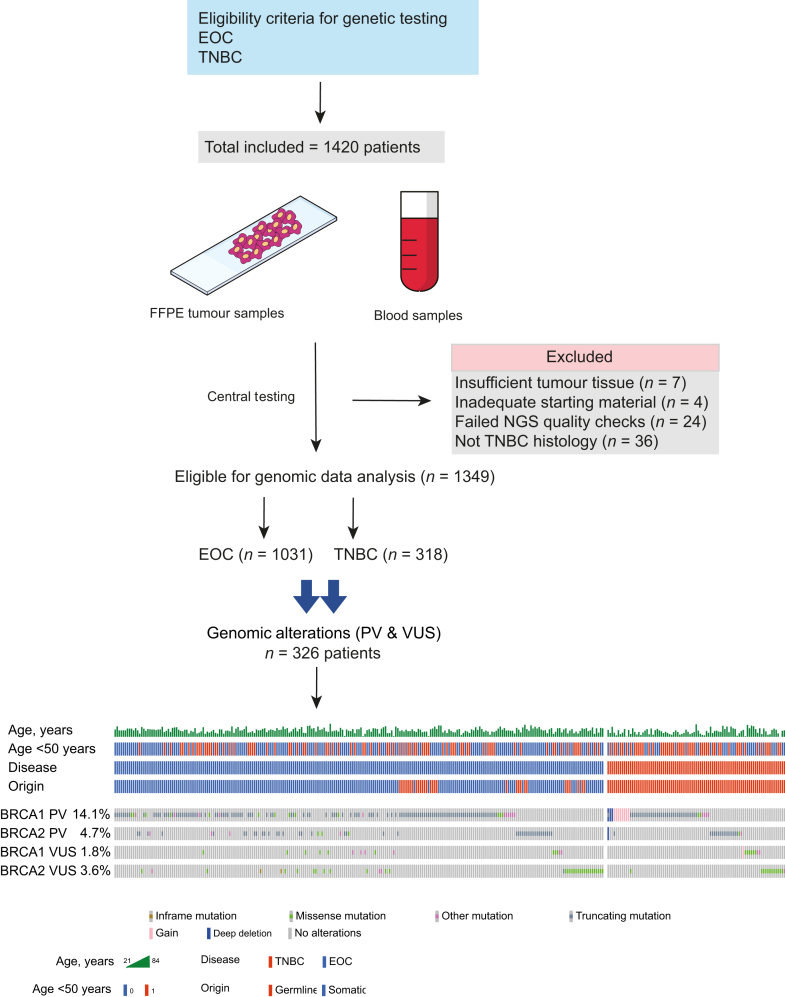
**Overview of the study design, inclusion and exclusion criteria**. Lower part is the oncoprint showing patients with clinically relevant variants (*n* = 326) including *BRCA1/2* pathogenic variants (PVs) and variants of uncertain significance (VUS) across our cohort and their baseline characteristics including age, disease [epithelial ovarian cancer (EOC) versus triple-negative breast cancer (TNBC)] and origin of sample (somatic versus germline). The oncoplot was split based on the disease with EOC to the left and TNBC to the right. NGS, next-generation sequencing.

Samples from 1420 patients were submitted for molecular analysis within the study. A total of 1349 patients completed at least one test and were included in the final analysis (Figure 1). The median age of the eligible cohort was 55 years (range 20-86 years) with one-third younger than 50 years (Table 1). All 318 patients with TNBC underwent g*BRCA* testing. Among 1031 patients with EOC, 121 (11.7%) had g*BRCA* only testing (Table 1). Molecular analysis was not successful for 36 patients with EOC. The causes were insufficient tumour tissue (*n* = 7), inadequate starting material (*n* = 4) and sequencing output failed quality metrics (*n* = 24) (Figure 1).

**Table 1:** Characteristics of the eligible patients in the study and the testing outcomes

	Total, *n* (%)	EOC, *n* (%)	TNBC, *n* (%)
Eligible patients	1420	1066	354
Successfully tested patients	1349	1031	318
Germline testing	439 (32.5)	121 (11.7)	318 (100)
Median age, years (range)	55 (20-86)	57 (20-86)	48 (20-83)
Age <50 years	455 (33.7)	285 (27.6)	170 (53.5)
*BRCA1/2* PVs	254 (18.8)	188 (18.2)	66 (20.8)
*BRCA1/2* VUS	72 (5.3)	21 (8.3)	51 (6.1)
*BRCA1* PVs	190 (14.1)	141 (13.7)	49 (15.4)
*BRCA2* PVs	64 (4.7)	47 (4.6)	17 (5.4)
*BRCA1* VUS	24 (1.8)	15 (1.5)	9 (2.8)
*BRCA2* VUS	48 (3.6)	36 (3.5)	12 (3.8)
*BRCA* PVs rate			
Age categories, years			
20 to <30	5 (13.9)	1 (5.6)	4 (22.2)
30 to <40	33 (24.6)	15 (22.1)	18 (27.3)
40 to <50	66 (23.1)	46 (23)	20 (23.3)
50 to <60	93 (22.3)	78 (23.3)	15 (18.1)
60 to <70	37 (10.9)	33 (11.3)	4 (8.3)
≥70	20 (14.7)	15 (12.6)	5 (29.4)

### Egyptian women with EOC/TNBC are enriched with BRCA1/2 pathogenic variants

**Figure 2:**
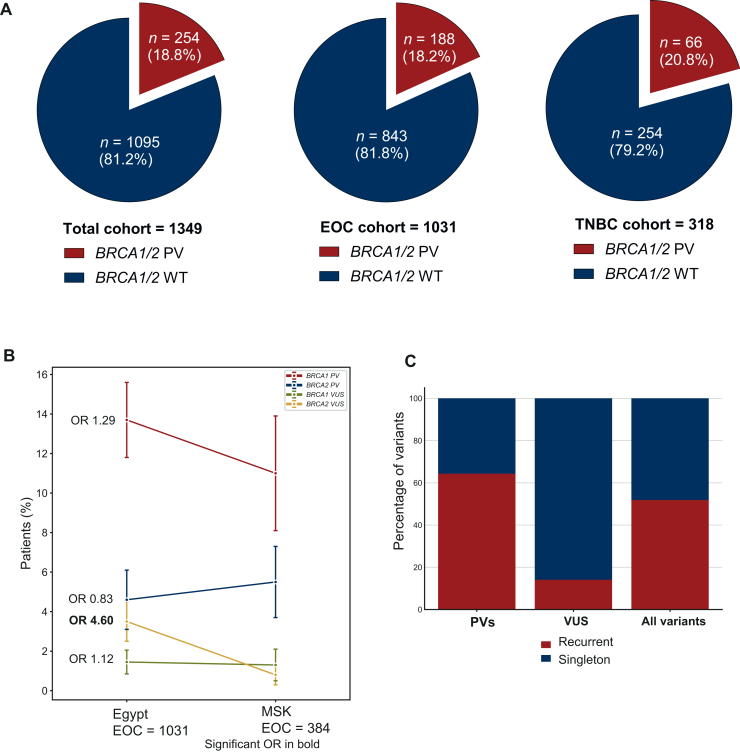
**Egyptian women with epithelial ovarian cancer (EOC)/triple-negative breast cancer (TNBC) enriched with pathogenic variants (PVs).** (A) Pie charts showing the rate of *BRCA1/2* PVs in the total cohort and EOC and TNBC subgroups. (B) Comparison of *BRCA* PVs and variant of uncertain interest (VUS) rate with the Memorial Sloan Kettering (MSK) cohort with EOC. The left bars depict percentages and 95% confidence intervals of the Egyptian EOC cohort (*n* = 1031) and the right bars depict the MSK cohort (*n* = 384); statistically significant odds ratios (ORs) are in bold. (C) Percentage of recurrent (occurred in two ore more patients) and singleton (occurred in one patient only) variants identified in the study. WT, wild-type.

We identified 343 clinically relevant variants. PVs or likely PVs totalled 258, affecting 254 patients (18.83%), and there were 85 VUS affecting 72 patients (5.34%) (Figure 2A). Across our cohort, two patients had co-occurrence of *BRCA1* and *BRCA2* PVs (Supplementary Figure S1, available at https://doi.org/10.1016/j.esmoop.2025.105510). The rate of *BRCA1/2* PVs in EOC and TNBC was 18.2% (*n* = 188) and 20.8% (*n* = 66), respectively.

We examined the difference between our cohort and a Western population cohort. The MSK EOC cohort was prospectively tested using the MSK-IMPACT panel and the pathogenicity assessment followed the ACMG guidelines (see Methods). No significant difference in the *BRCA1/2* PVs was observed between the Egyptian EOC and the MSK EOC cohorts (Figure 2B). It is noteworthy that the Egyptian EOC cohort was significantly enriched with *BRCA2* VUS [OR 4.6, 95% confidence interval (CI) 1.41-15.01] (Figure 2B).

### Patterns of pathogenic variants and their lineage uniqueness

The PVs were significantly enriched with recurrent variants compared with VUS (64.34% versus 14.11%, *P* < 0.001) (Figure 2C). The majority of PVs were truncating mutations (either frameshift or nonsense variants, constituting 78.2% and 84.6% of the PVs in *BRCA1* and *BRCA2*, respectively) (Supplementary Figure S2, available at https://doi.org/10.1016/j.esmoop.2025.105510). The most commonly recurrent PVs were *BRCA1* V409∗ (*n* = 21, 8.27%), *BRCA1* C1146Lfs∗ (*n* = 16, 6.30%), *BRCA1* Q1227∗ (*n* = 8, 3.15) and *BRCA1* Q1111Nfs∗ (*n* = 6, 2.36%) (Supplementary Table S1, available at https://doi.org/10.1016/j.esmoop.2025.105510). Three of these top recurrent variants occur in exon 11 of *BRCA1* gene and are caused by a deletion of one (V409∗) or four nucleotides (C1146Lfs∗ and Q1111Nfs∗). None of these are reported in normal populations (genomAD). Although the rate of PVs in our cohort was similar to the Western cancer population, there was a significant difference in the enrichment at variant level. Of the top 10 recurrent *BRCA1/2* PVs, only 3 PVs were reported in the two large western datasets, the MSK (*n* = 1610) and the UK diagnostic laboratories cohorts (*n* = 80 722) (Supplementary Table S1, available at https://doi.org/10.1016/j.esmoop.2025.105510).

We examined the lineage-uniqueness of variants across *BRCA1* and *BRCA2* in ovarian versus breast cancer (Supplementary Figure S3A and B, available at https://doi.org/10.1016/j.esmoop.2025.105510). We identified 12 recurrent *BRCA2* PVs affecting 31 patients. Interestingly, the majority (26/31, 84%) of recurrent *BRCA2* PVs were lineage-unique, with only two PVs (affecting five patients) recurrent in both lineages, i.e. ovarian and breast cancer (Supplementary Figure S3A and B, available at https://doi.org/10.1016/j.esmoop.2025.105510) (chi-Square goodness of fit, *P* = 0.00225). The most common ovarian-unique variants were *BRCA2* V1283Kfs∗ and R2272fs (Supplementary Figure S3B, available at https://doi.org/10.1016/j.esmoop.2025.105510). On the other hand, we identified 30 recurrent *BRCA1* PVs affecting 130 patients, and only 36% (83/130, 64%) were lineage-unique and 14 PVs were recurrent across both lineages (chi-square goodness of fit, *P* = 0.19).

### Outcomes of BRCA1/2 testing in EOC cohort

A total of 1031 patients with EOC had *BRCA1/2* testing, either germline or somatic. The median age of the EOC cohort was 57 years (range 20-86 years). A total of 285 (28%) patients were aged <50 years. The rate of *BRCA1/2* PVs was 18.33% (*n* = 188), with 13.7% (*n* = 141) occurring in *BRCA1* and 4.6% (*n* = 47) occurring in *BRCA2* (Figure 1B and Table 1). The most common type of PV in the EOC was truncating mutation (*n* = 155, 82.01%). The missense and splice variants occurred in 19 (10.05%) and 15 (7.94%) women, respectively. The most common PVs were *BRCA1* V409∗ (*n* = 15, 7.98%), *BRCA1* C1146Lfs∗ (*n* = 12, 6.38%) and *BRCA1* Q1227∗ (*n* = 7, 3.72%). Seventy-one PVs were singletons (occurred once only) and were detected in 37.7% of women harbouring *BRCA1/2* PVs. The majority of EOC patients had somatic testing, with a subgroup of only 121 (11.7%) patients having germline testing. The rate of *BRCA1/2* PVs was similar in the germline subgroup compared with the somatic testing group (19% versus 18.1%).

**Figure 3:**
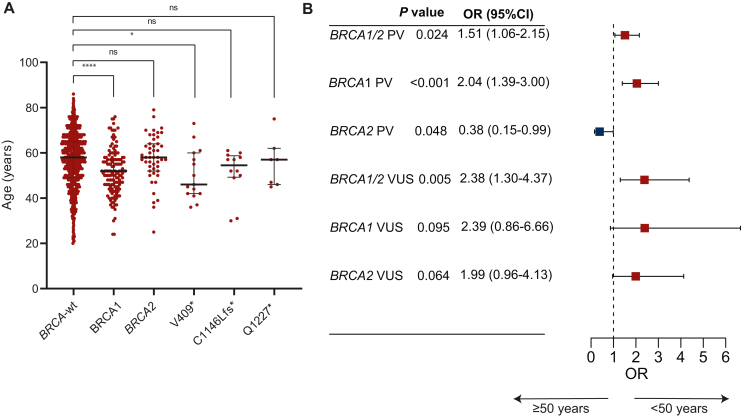
***BRCA1/2* and age of onset.** (A) The age distribution of patients with *BRCA1* and *BRCA2* pathogenic variants (PVs) compared with wild-type. Also included separately the top three recurrent *BRCA1* variants. (B) Forest plot of the odds ratio (OR) and 95% confidence interval (CI) of *BRCA1/2* PV and variants of uncertain interest (VUS) in women age categorized at 50 years.

We examined the association of age at diagnosis with *BRCA1/2* PV status in the EOC cohort. Patients with *BRCA1* PVs had a significantly younger median age at diagnosis compared with *BRCA1/2* wild-type subgroup (Figure 3A and B). This was not evident with *BRCA2* PVs. Interestingly, the top recurrent PV, *BRCA1* V409∗, was associated with the youngest age (Figure 3A). The rates of *BRCA1/2* PVs and VUS were not statistically significant across different age groups (Supplementary Figure S4, available at https://doi.org/10.1016/j.esmoop.2025.105510). In addition, we ran logistic regression analysis using age 50 years as cut-off. *BRCA1* PVs were more likely to occur at younger age (<50 years) (OR 2.04, *P* < 0.001), while *BRCA2* PVs were less likely to occur at younger age (OR 0.38, *P* = 0.048) (Figure 3B). There was a trend of *BRCA1* and *BRCA2* VUS to occur at younger age (Figure 3B).

### Outcomes of germline testing of TNBC cohort

Germline genetic testing was successfully carried out for a total of 318 patients with TNBC. The median age was 48 years (range 22-71 years). Sixty-seven (21%) of patients harboured germline PVs in *BRCA1/2* (Table 1). The frequency of PVs in *BRCA1* was significantly higher than in *BRCA2* [49 patients (15%) versus 17 (5%) patients, *P* < 0.0001]. Patients with *BRCA1 PVs* were significantly younger, with a median age of 43 years versus 49 years (*P* = 0.0.003), and no significant difference in the median age was observed in *BRCA2* PV carriers (51 years versus 47.5 years, *P* = 0.051). Patients with *BRCA1/2* VUS did not significantly differ in median age from patients with *BRCA* wild-type (54 years versus 48 years, *P* = 0.47).

### Egyptian patients are enriched with intron VUS and fewer missense variants

The ClinVar database hosted at the United States National Library of Medicine largely depends on submissions from the Western population. We compared the frequency of VUS class types in ClinVar with our current cohort. We extracted 12 391 VUS reported in ClinVar. The most common variant type in the ClinVar database was missense (*n =* 11 214, 90.5%). Our cohort was significantly enriched with intron variants compared with ClinVar VUS (16.5% versus 4.4%, *P* < 0.0001) (Supplementary Figure S5, available at https://doi.org/10.1016/j.esmoop.2025.105510). Meanwhile, our cohort was less enriched with missense variants (76.5% versus 90.5%, *P* < 0.0001). Splice variants showed no significant difference (1.18% versus 0.83%, *P* = 0.72). There was no significant difference in other variant classes (*P* > 0.05) (Supplementary Figure S5, available at https://doi.org/10.1016/j.esmoop.2025.105510).

### Experimental and computational reclassification of VUS

**Figure 4:**
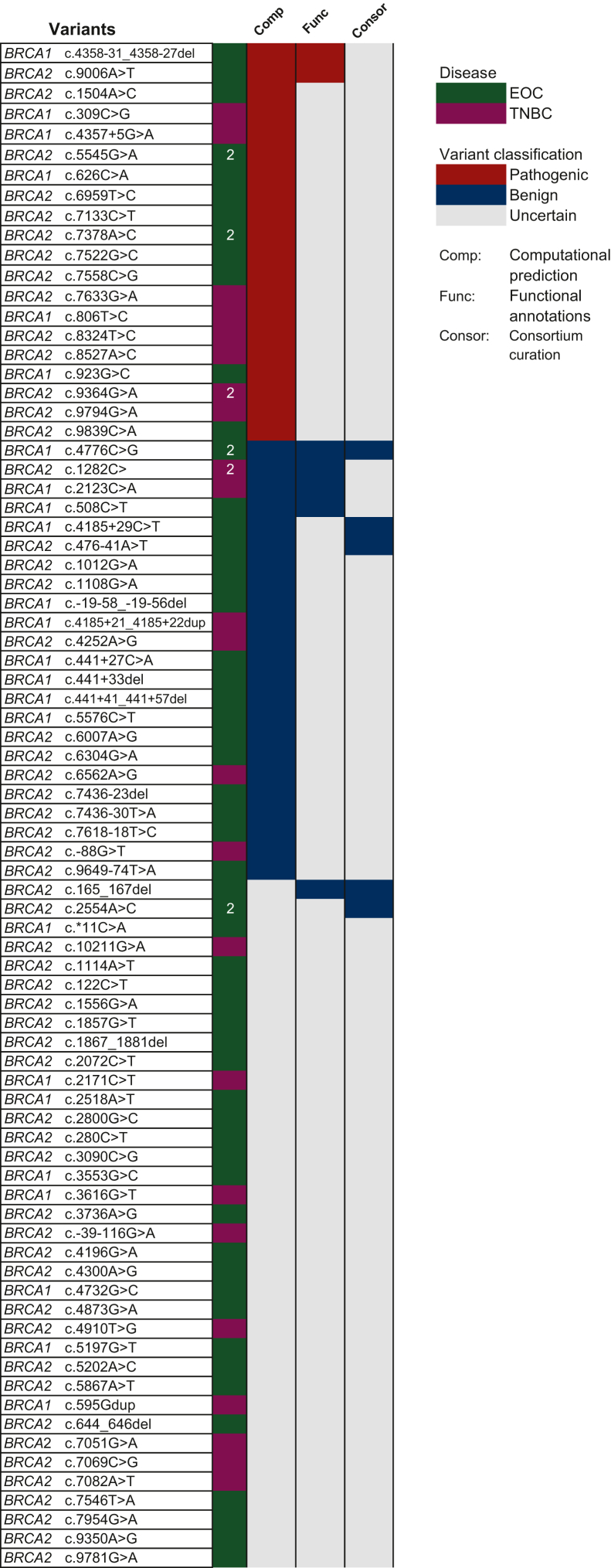
**Variants of uncertain interest (VUS) reclassification.** List of the VUS and their reclassification status based on computational prediction and functional data. Green colour represents epithelial ovarian cancer (EOC) and magenta represents triple-negative breast cancer (TNBC); variants occurred in two patients indicated by number ‘2’. VUS reclassified as pathogenic represented with red colour, blue for benign reclassification and grey for VUS that remained uncertain.

We undertook a systematic approach to reclassify the identified VUS in our cohort (*n* = 85 variants) (Figure 4). We adopted a combined approach including computational prediction, functional annotations from high-quality experimental data and academic consortia curation to reclassify the identified VUS (see Methods). Using academic consortia curation, namely ClinVar and Can-Var, five variants were reclassified as benign including *BRCA2* c.165_167del and c.476-41A>T. The majority remained of uncertain significance. No large-scale healthy control data are available for the Egyptian population. The majority (68%, 58/85) of the identified VUS were not reported in the GenomAD population. We ran a computational pipeline (MVP) for missense VUS variants in our cohort (Figure 4). A total of 18 missense VUS (affecting 21 patients) were candidate pathogenic variants, 11 missense VUS (affecting 13 patients) were candidate benign variants. Variants with borderline scores or that failed to be scored remained VUS (Supplementary Figure S6, available at https://doi.org/10.1016/j.esmoop.2025.105510).

We conducted a systematic review of the literature for functional and experimental analysis of these variants. High-quality data defined by the cancer variant interpretation group (Can-VIG UK) were available for seven variants affecting nine patients, and they were all concordant with the computational predictions (Figure 4). This combined approach achieved a reclassification rate of 52.3% (34/65). Similarly, we ran SpliceAI for the intron variants (*n* = 15). Two variants were predicted to lead to loss of splice region site and are considered candidate pathogenic, and the remaining 12 were shown to have no impact on the splicing except one variant with a borderline score considered uncertain.

Taken together, our combined approach provides a reclassification of 45 variants (affecting 60% of patients). Our reductionist reclassification approach is feasible in limited resource settings in the context of the lack of case-control and genetic epidemiology data.

## Discussion

To our knowledge, this is the largest study to provide comprehensive *BRCA1/2* testing for Egyptian patients with EOC or TNBC. For the first time, we were able to provide a reliable prevalence of *BRCA1/2* mutations in these diseases with a thorough dissection of the patterns of PVs. Additionally, we could provide a limited resource-conscious approach to gain insights into the pathogenicity of VUS.

We could verify some recurrent PVs in the Egyptian series that differ from other ethnicities. For example, the V409∗ variant was the most common PV in our series. The *BRCA1* V409∗ variant was initially reported in the Japanese population^16^ but was recently reported in the Arab population.^17^^,^^18^ The *BRCA1* Q1111Nfs∗ is an internationally distributed founder mutation, with a common ancestral origin recently attributed to Iberia.^19^ The fact that these recurrent mutations affect *BRCA1* exon 11 may have therapeutic relevance because there is evidence that tumours that harbour *BRCA1* exon 11 mutations can derive platinum and poly (ADP-ribose) polymerase (PARP) inhibitor resistance through generation of an exon 11-skipped hypomorphic isoform that partially restores *BRCA1* function.^20^ Notably, the three Ashkenazi Jewish pathogenic founder mutations (*BRCA1* 185delAG, *BRCA1* 5382insC and *BRCA2* c.5946del) were not detected in our cohort.

One important aspect we tried to explore is the lineage-unique PVs. We observed that the majority of the identified *BRCA2* PVs were specific to one disease (either EOC or TNBC). This may be of importance when counselling the *BRCA1/2* carriers about possible risk reduction strategies. Several nomograms are being developed to predict the future risk of having breast cancer, contralateral breast cancer or EOC in women with *gBRCA* mutations depending on several factors, one of them being the type of PV in *BRCA1/2* gene. With the scarce data from Northern African or Egyptian women, our data could provide a deeper insight into this aspect.

The *BRCA1/2* PV prevalence showed similarity in age distribution compared with the Western series.^11^ Specifically, our findings were consistent with other studies showing that *BRCA1*-mutant EOC is associated with younger age; meanwhile, *BRCA2*-mutant is not.^21^^,^^22^ This was observed, despite the significantly lower age of incidence (and lower life expectancy) in Egypt.

Limited resource settings suffer from a lack of genetic epidemiology, case-control, segregation and phenotype data, which makes VUS interpretation challenging. We showed that by adopting a reductionist approach relying on computational prediction and functional annotations, it is feasible to reclassify up to 60% of the identified VUS.

One of the key strengths of this study is applying whole gene sequencing and not limited to specific hotspot regions of the *BRCA1/2* genes. This allows us to identify splice site, UTR and intronic variants. We believe that a significant pool of clinically relevant variants would have been missed if a restricted hotspot panel had been applied.

One of the limitations of this study is the lack of treatment outcomes. As in other limited-resource settings, PARP inhibitors are not reimbursed in the public health care system in Egypt. However, our findings helped to delineate the rate of patients who likely will benefit from precision-oncology-based management. Our VUS reclassification approach should be used cautiously in settings where there is lack of genetic epidemiology and case-control phenotype data with ongoing auditing and validation. Recently, the ongoing Egypt Genome project has been launched, aiming to sequence 100 000 healthy Egyptian individuals and 8000 individuals with disease, including top prevalent malignancies.^23^ The project aims to build an Egyptian genome reference that encompasses the genetic variants among Egyptians. These data will be valuable to validate our preliminary findings once available in the near future.

Our results will guide the integration of NGS-based testing into the clinical management of patients with breast and ovarian cancer, especially those from understudied ancestries. Furthermore, the findings we derived from whole gene analysis will inform the design of focused initial screening genetic testing. Our cohort is enriched with intronic variants. Again, this carries important implications against the restricted approach of testing exonic or hotspot regions of *BRCA1/2,* which might lead to missing significant numbers of splice pathogenic and intronic VUS variants. Overall, we believe that this work is a significant step towards harnessing the full benefits of NGS-based genetic testing to transform the precision oncology of patients with limited access to molecular testing resources.
