# Supplementary material for: Genetic testing for BRCA1/2 variants in Northern African women with ovarian and breast cancers: a multicentre study of an under-represented ancestry
Source: ESMO Open. 2025 Jul 25;10(8):105510. doi: 10.1016/j.esmoop.2025.105510 (PMC12314369; doi:10.1016/j.esmoop.2025.105510)

Supplementary Information File

Genetic testing for *BRCA1/2* variants in Northern African women with ovarian and breast cancers: A multicentre study of an underrepresented ancestry.

Shohdy KS et al.,

Supplementary Tables

Supplementary Table 1: The frequency of the top 10 recurrent *BRCA1/2* PVs in Egyptian cohort and their occurrence in the UK Diagnostic Labs (NDRS) and the Memorial Sloan Kettering Cancer Centre (MSK) cohorts.

Gene	c.HGVS	p.HGVS	cases.Egypt	total.Egypt	cases.NDRS	total.NDRS	cases.MSK	total.MSK
BRCA1	c.1224delA	V409*	21	1349	0	80722	0	1610
BRCA1	c.3436_3439del	C1146Lfs*8	16	1349	0	80722	0	1610
BRCA1	c.3679C>T	Q1227*	8	1349	0	80722	1	1610
BRCA1	c.3331_3334del	Q111Nfs*	6	1349	96	80722	0	1610
BRCA1	c.4485-1G>A	c.4485-1G>A	5	1349	0	80722	0	1610
BRCA1	c.5030_5033del	T1677fs	5	1349	7	80722	0	1610
BRCA1	c.5074G>C	D1692H	5	1349	0	80722	0	1610
BRCA1	c.5095C>T	R1699W	5	1349	0	80722	0	1610
BRCA2	c.3847_3848del	V1283Kfs*2	4	1349	0	80722	0	1610
BRCA2	c.6814del	R2272fs	4	1349	0	80722	0	1610

Supplementary Figure 1: Plot of the 14 patients with multiple variants, grey color indicates affection, the number in bold indicates the count of variants in each category. Origin of the sample either somatic or germline indicated by blue and red colors, respectively. Two patients had multiple PVs in *BRCA1* and *BRCA2*, one patient with TNBC had a missense VUS (*BRCA2* c.7051G>A) co-occurred with a pathogenic *BRCA1* p.Cys1146fs mutation. The remaining 11 patients had VUS in the same gene with the majority (10/11) in *BRCA2*.

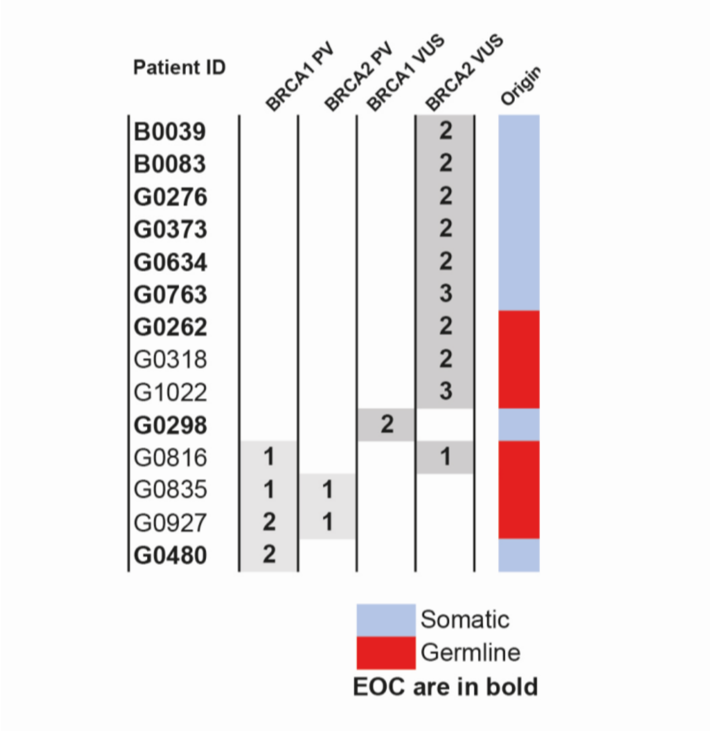

Supplementary Figure 2: Percentages of variant classes of identified PVs and VUS in *BRCA1* and *BRCA2* across our cohort.

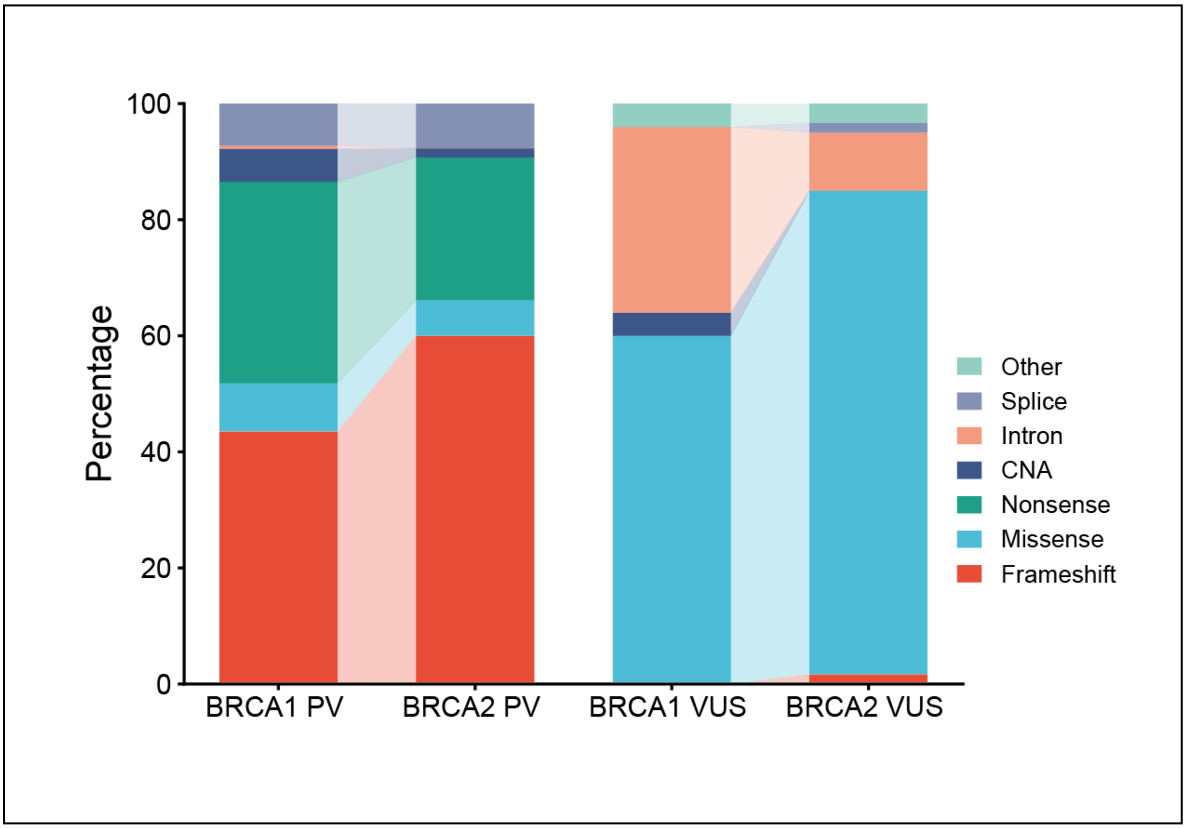

Supplementary Figure 3: Lollipops for *BRCA1 (A)* and *BRCA2 (B)* genes with PVs for EOC (above) and TNBC (below). Numbers inside the circles are number of patients harboured the variant. The majority (84%) of the recurrent *BRCA2* PVs were not shared across EOC and TNBC subgroups.

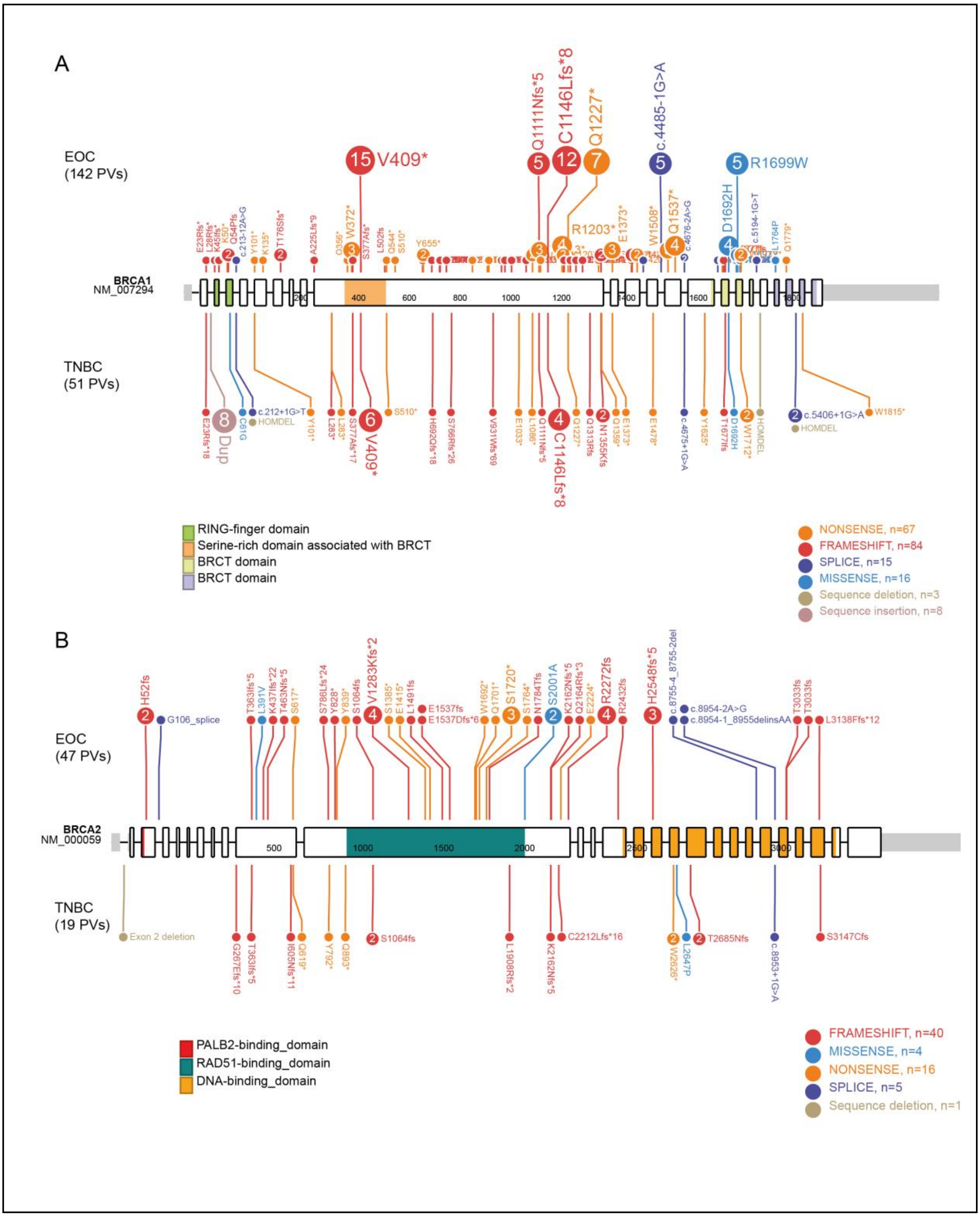

Supplementary Figure 4: Frequency of *BRCA1/2* PVs and VUS across various age groups.

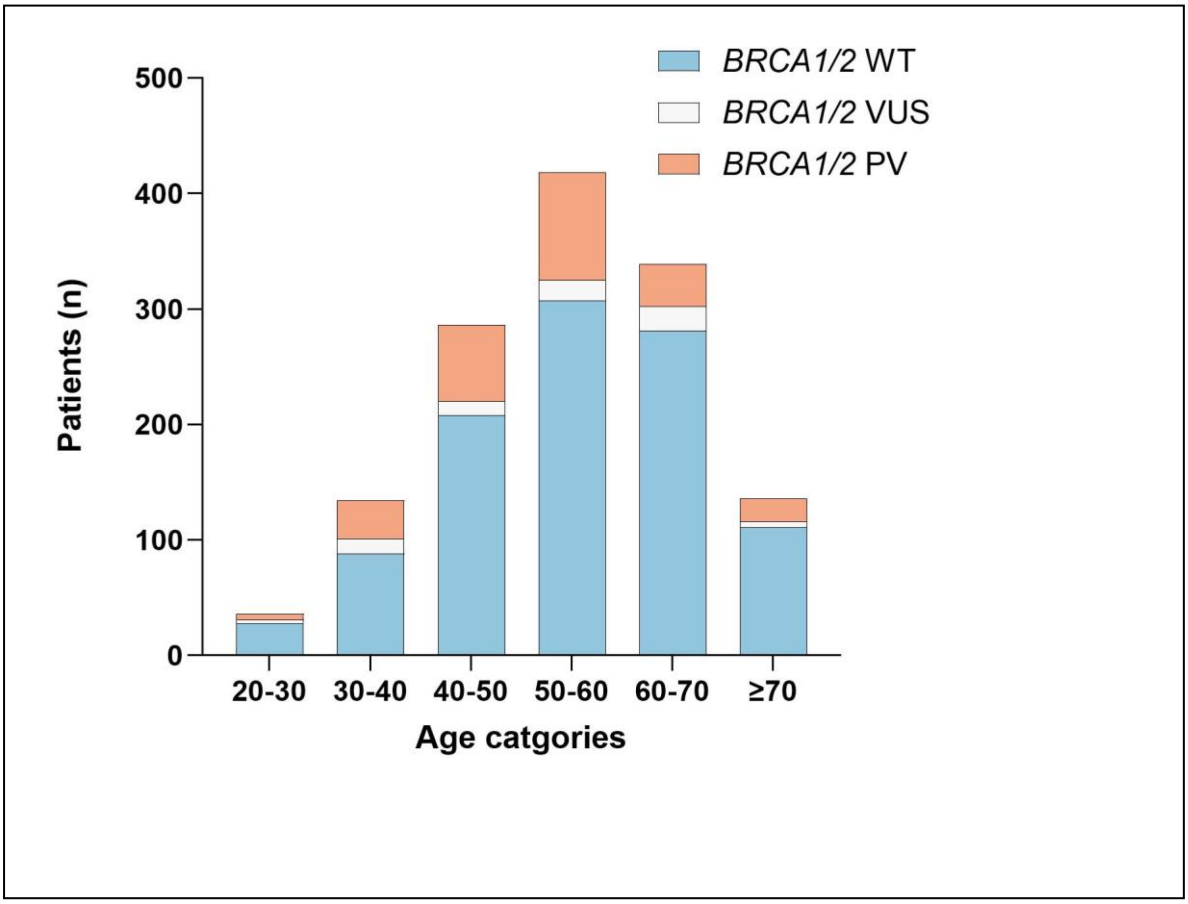

Supplementary Figure 5: VUS in the Egyptian cohort enriched with intronic variants in comparison to the ClinVar VUS.

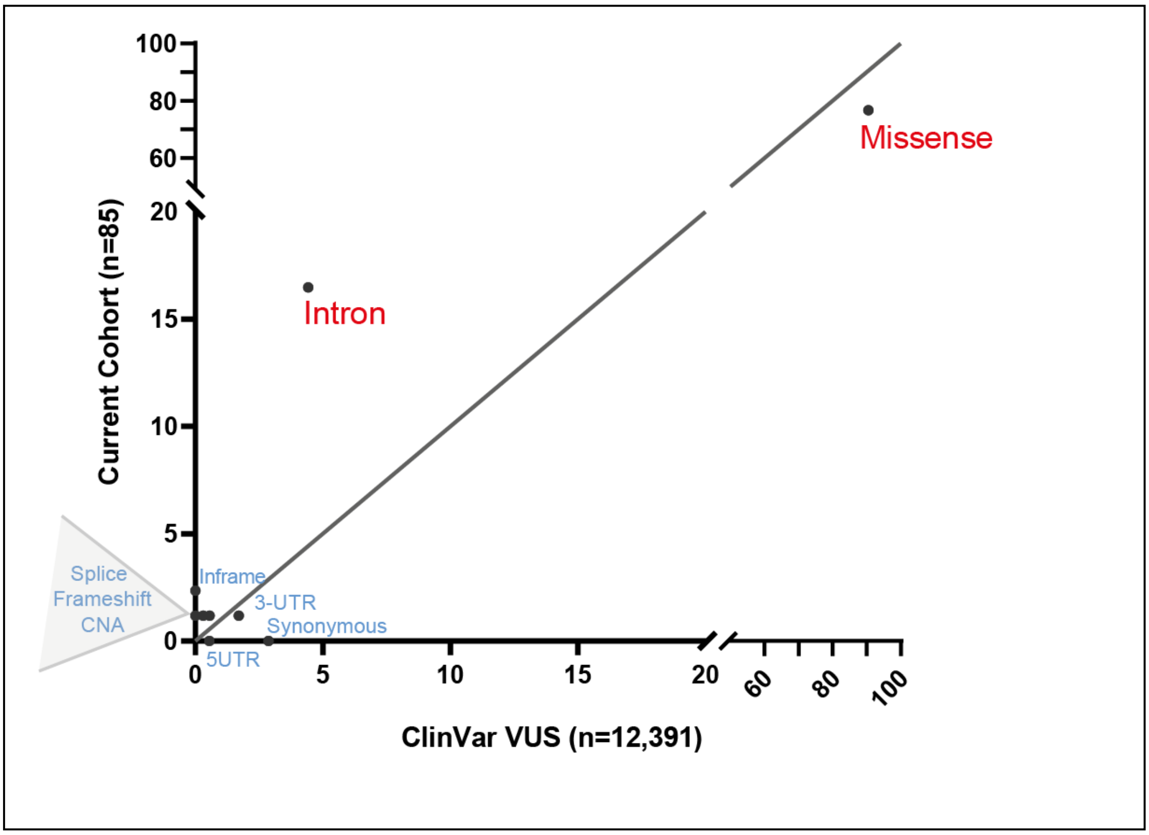

Supplementary Figure 6: Bubble plot showing the MVP scores of the missense VUS variants in our cohort. The vertical line indicates candidate pathogenic cutoff at 0.85. Candidate pathogenic variants are shaded in purple. *BRCA1* variants in light brown and *BRCA2* variants in grey.

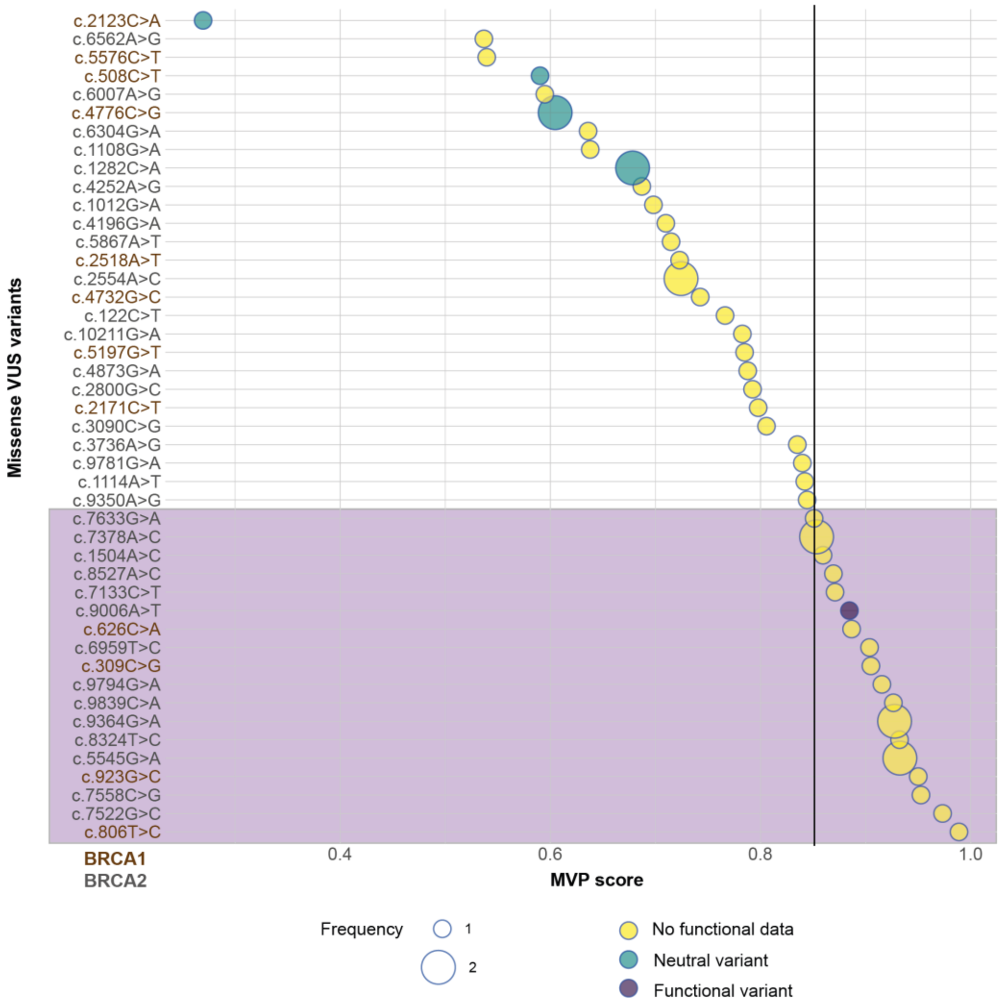

Supplement: Supplementary Material [file mmc1.pdf]
